# Association of the G134A and G184C Polymorphisms in the *CYP1A1* Gene with Lung Cancer Incidence

**DOI:** 10.5487/TR.2008.24.2.109

**Published:** 2008-06-01

**Authors:** Doug-Young Ryu, Mingai Huang, Changbo Park, Soo Im Chang, Ruth Im, Seong-Jin Choi, Na-Young Kim, In Won Park, Byoung Whui Choi, Jae Yeol Kim, Jong Wook Shin, Jae Chul Choi, Byung-Sun Choi, Jung-Duck Park

**Affiliations:** 13 College of Veterinary Medicine, Seoul National University, Seoul, 151-742 Korea; 23 College of Medicine, Chung-Ang University, Seoul, 156-756 Korea

**Keywords:** *CYP1A1*, Single nucleotide polymorphism, G134A, G184C, Gly45Asp, Ala62Pro, Lung cancer

## Abstract

The G184C and G134A single nucleotide polymorphisms (SNPs) of the *CYP1A1* gene result in Ala62Pro and Gly45Asp substitutions, respectively. Here, we tested whether these SNPs are associated with an alteration in lung cancer incidence. We examined 80 Korean subjects with lung cancer and 240 age- and sex-matched controls. For each subject, the *CYP1A1* gene was PCR amplified and sequenced. We observed that the odds ratio (OR) for lung cancer was 3.37 higher in subjects with the G184C polymorphism than in controls (95% confidence interval (CI), 0.89~12.73, *P* = 0.07). In contrast, the OR for lung cancer was 1.23 in subjects with the G134A polymorphism compared to controls (95% CI, 0.68~2.20, *P* = 0.49). The G184C polymorphism exacerbated the effects of smoking on lung cancer development. Gene-smoking interaction analyses revealed that past or present smokers with the G184C polymorphism had a higher incidence of lung cancer (OR, 24.72; 95% CI, 4.48~136.31; *P* < 0.01) than control smokers (OR, 6.65; 95% CI, 2.72~16.28; *P* < 0.01). However, there was only a slight difference in the ORs for lung cancer between control smokers and smokers with the G134A polymorphism. These findings suggest that the G184C polymorphism, but not the G134A polymorphism, is associated with an increased risk of lung cancer.

## INTRODUCTION

The CYP1A1 enzyme is a member of the human cytochrome P-450 family. It acts by metabolizing xenobiotics including the tobacco carcinogen benzo[*a*]pyrene and other polycyclic aromatic hydrocarbons to mutagenic epoxide and diols [e.g. benzo[*a*]pyrene-diol-epoxide] (McManus *et al.*, 1990; Bartsch *et al.*, 2000). To date, more than 17 nucleotide polymorphisms including at positions such as - 3229, - 3219, 134, 184, 233, 518, 1413 (SNP ID: rs4987133), 1636, 2414, 2453, 2455, 2461, 2500, 2515, 2546, 3205, and 3801 and a frameshift mutation (due to a single base insertion between 2346 and 2347) in the *CYP1A1* gene have been reported (Spurr *et al.*, 1987; Hayashi *et al.*, 1991; Crofts *et al.*, 1993; Cascorbi *et al.*, 1996; Smart *et al.*, 2000; Chevalier *et al.*, 2001; Saito *et al.*, 2002; Park *et al.*, 2004, Solus *et al.*, 2004).

Because of its metabolic role in the production of active carcinogens, it is not surprising that *CYP1A1* polymorphisms have been linked with lung cancer. Two of the best understood polymorphisms include a transition of T3801C in the 3' non-coding region, which results in the introduction of an MspI restriction site and a transition of A2455G, which results in an Ile to Val change. These SNP mutations have been associated with an increased risk of lung cancer in various ethnic groups (Song *et al.*, 2001; Sugimura *et al.*, 1998; Hung *et al.*, 2003; Vineis *et al.*, 2003).

The G184C and G134A polymorphisms of *CYP1A1* result in Ala62Pro and Gly45Asp substitutions, respectively, and were first identified in Korean and Japanese populations, respectively (Chevalier *et al.*, 2001; Saito *et al.*, 2002). However, the functional role of these polymorphisms in cancer susceptibility has not been addressed. In the present study, we have tested the effects of these two polymorphisms and their relationships with smoking in terms of the risk of lung cancer incidence.

## MATERIALS AND METHODS

***Study subjects.*** Our study included 80 lung cancer patients between 30 and 91 years of age that were admitted to Chung-Ang University Hospital (Seoul, Korea) between 2005 and 2007. For each patient, three controls were randomly selected by matching sex and ages, were ± 3 years of age of the patients, and had not been diagnosed with lung cancer. All subjects gave written informed consent to participate in this study. This study was approved by the Ethics Committee for the Protection of Persons and Animals in Biochemical Research at the Institute of Medical Science, Chung-Ang University (Seoul, Korea).

***Genotyping analysis.*** Genomic DNA extracted from the blood from each participating individual was amplified by polymerase chain reaction (PCR). Primers for the PCR were designed to produce DNA fragments encompassing Gly45 and Ala62 (Accession #X02612). The primer sequences were 5'-CCA ATC TGA CGG CTT GAC TT-3' and 5'-CCC ATG CAG TTC CTC TTA CC-3'. PCR was performed for 35 cycles at 94°C for 1 min, 59°C for 1 min, and 72°C for 1 min. After amplifications, PCR products were analyzed by DNA sequencing using a 5'-CCG GCC CTT GAA ATC ATC-3' primer.

Linkage analysis between the G184C and G134A polymorphisms was performed for the subjects who had both polymorphisms. The PCR products were cloned into the TA vector pGEM-T Easy system (Promega, Madison, WI), and the individual clones were sequenced using the T7 primer.

***Statistical analysis.*** The odds ratios (ORs) and 95% confidence intervals (CIs) were analyzed in a conditional logistic regression model using the PhReg procedure in SAS version 10.1 (SAS Institute, Cary, NC).

## RESULTS AND DISCUSSION

The defining characteristics of the study subjects are shown in Table 1. A total of 248 males and 72 females participated in this study. The average age of the lung cancer patients and controls were 66.3 ± 10.7 and 65.6 ±

**Table 1.** Demographic characteristics of study subjects

| Characteristics | Number of patients | Number of controls | OR (95% CI) | p value |
|---|---|---|---|---|
| Subjects | 80 | 240 | | |
| Gender | | | | |
| Male | 62 | 186 | | |
| Female | 18 | 54 | | |
| Age (mean in years ± S.D.) | 66.3 ± 10.7 | 65.6 ± 9.9 | | |
| Smoking | | | | |
| Non-smoker | 17 | 110 | 1.00 | |
| Smoker (past or present) | 63 | 130 | 6.93 (2.83~16.98) | < 0.01 |

**Table 2.** Association between lung cancer incidence and the G134A and G184C polymorphisms in the *CYP1A1* gene

| Subjects | Number of patients | Number of controls | OR (95% CI) | p value |
|---|---|---|---|---|
| Without G134A | 56 | 177 | 1.00 | |
| G184C/WT | 3 | 2 | | |
| WT/WT | 53 | 175 | | |
| With G134A | 24 | 63 | 1.23 (0.68~2.20) | 0.49 |
| G134A/G134A | 1 | 3 | | |
| G134A/WT | 21 | 57 | | |
| G134A/G184C | 2 | 3 | | |
| Without G184C | 75 | 235 | 1.00 | |
| G134A/G134A | 1 | 3 | | |
| G134A/WT | 21 | 57 | | |
| WT/WT | 53 | 175 | | |
| With G184C | 5 | 5 | 3.37 (0.89~12.73) | 0.07 |
| G134A/G184C | 2 | 3 | | |
| G184C/WT | 3 | 2 | | |

**Table 3.** Effects of either the G134A or G184C polymorphism of *CYP1A1* and smoking on the risk of developing lung cancer

| Subject | Smoking | Number of patients | Number of controls | OR (95% CI) | p value |
|---|---|---|---|---|---|
| Without G134A | No | 14 | 87 | 1.00 | |
| With G134A | No | 3 | 23 | 0.92 (0.24~3.47) | 0.90 |
| Without G134A | Yes | 42 | 90 | 6.48 (2.48~16.92) | < 0.01 |
| With G134A | Yes | 21 | 40 | 7.91 (2.67~23.43) | < 0.01 |
| Without G184C | No | 17 | 109 | 1.00 | |
| With G184C | No | 0 | 1 | - | - |
| Without G184C | Yes | 58 | 126 | 6.65 (2.72~16.28) | < 0.01 |
| With G184C | Yes | 5 | 4 | 24.72 (4.48~136.31) | < 0.01 |

Subjects with and without each polymorphism were separated according to smoking status.

9.9, respectively. Smoking was associated with a 6.93-fold increased risk of lung cancer (95% CI, 2.83~16.98; $P < 0.01$).

The distribution of the *CYP1A1* polymorphisms among all participants is shown in Table 2. Allelic frequencies of G134A and G184C polymorphisms were 14.2% (91 alleles) and 1.6% (10 alleles) among the 320 subjects studied (i.e., 640 alleles), respectively, and no linkage between them was observed. The frequency of allelic combination between the two polymorphisms was 1.6% (5 among 320 subjects).

The G134A polymorphism was observed in 24 cancer patients (30%). One homozygote was found among them. Of those 23 heterozygous carriers, 2 patients had both the G134A and G184C polymorphisms (designated G134A/G184C). A slightly lower percentage of controls (26.3%) carried the G134A polymorphism. This polymorphism was found in both alleles in 3 of the controls and was present in 1 copy for the remaining 60 individuals. Three control individuals carried both the G134A and G184C polymorphisms. The group of subjects without the G134A polymorphism was set as baseline (OR = 1.00). The OR for subjects with at least one G134A-containing allele was 1.23 (95% CI, 0.68~2.20; $P = 0.49$). Statistical analyses demonstrated that there was no significant association between the G134A polymorphism and lung cancer risk.

In contrast, the G184C polymorphism was detected in 5 cancer patients and 5 controls. None of the individuals in this study were homozygous for the G184C polymorphism. If the subjects lacking the G184C polymorphism were set at an OR = 1.00, then the OR for lung cancer in subjects with the G184C polymorphism was 3.37 (95% CI, 0.89~12.73; $P = 0.07$). The data indicate that subjects with the G184C polymorphism have a near-significantly increased risk of lung cancer.

To investigate whether any gene-environment interactions exist, the subjects were further divided according to their smoking status (Table 3). The OR for smokers containing one of these polymorphisms was calculated using non-smoker subjects lacking these polymorphisms as a baseline (OR = 1.00). The G134A polymorphism only slightly increased lung cancer risk among the subjects with a current or previous history of smoking. These smokers with at least one G134A-containing allele had an OR of 7.91 (95% CI, 2.67~23.43; $P < 0.01$), whereas those lacking this polymorphism had an OR of 6.48 (95% CI, 2.48~16.92; $P < 0.01$).

In contrast, smokers with a G184C-containing allele had an OR of 24.72 (95% CI, 4.48~136.31; $P < 0.01$), whereas those smokers lacking this polymorphism had an OR of 6.65 (95% CI, 2.72~16.28; $P < 0.01$). This demonstrates an increased risk for the smokers with the G184C polymorphism in developing lung cancer.

In summary, our findings suggest that it is unlikely that the G134A polymorphism influences lung cancer risk. However, individuals with the G184C polymorphism, especially those with a history of smoking, have an increased risk of acquiring lung cancer. Additional studies will be required to evaluate the effects of these polymorphisms on the metabolism of xenobiotics and their roles in carcinogenesis.

## ACKNOWLEDGEMENTS

This work was supported by Korea Research Foundation Grant KRF 2005-E00046.

## Abbreviations

CYP: cytochrome P-450
CI: confidence interval
OR: odds ratio
PCR: polymerase chain reaction
SNP: single nucleotide polymorphism
WT: wild-type
